# Congenital nasal pyriform aperture stenosis: A rare cause of nasal airway obstruction in a neonate

**DOI:** 10.4103/0971-3026.73539

**Published:** 2010-11

**Authors:** Elsa M Thomas, Sridhar Gibikote, Jyoti S Panwar, John Mathew

**Affiliations:** Department of Radiology, Christian Medical College, Vellore, Tamil Nadu, India; 1 Department of ENT and Head and Neck Surgery, Christian Medical College, Vellore, Tamil Nadu, India

**Keywords:** Choanal atresia, CNPAS, holoprosencephaly, megaincisor, pyriform aperture stenosis

## Abstract

Congenital nasal pyriform aperture stenosis (CNPAS) is a rare cause of nasal airway obstruction that clinically mimics choanal atresia, but needs to be differentiated from the latter because of the widely divergent modes of management. We present a case of CNPAS, to highlight the importance of recognizing the classic signs of CNPAS on cross-sectional imaging.

## Introduction

Congenital nasal pyriform aperture stenosis (CNPAS), first clinically described in 1989,[1] is a rare cause of neonatal nasal airway obstruction. It typically presents with clinical features that may mimic posterior choanal atresia,[2] and it is important to differentiate it from the latter as there are differences in patient management.[3]

## Case Report

A 30-day-old female baby, born at full-term, to nonconsanguinous parents, presented with a history of feeding difficulty and failure to thrive. There was a history of respiratory distress and cyanosis at birth. The antenatal period was uneventful. Clinical examination revealed dysmorphic features, with microcephaly, a cone-shaped occiput, microphthalmia, proptosis, bilateral simian crease, and a depressed nasal bridge. The child was noted to have mouth-breathing. There was no evidence of a cleft palate. A flexible nasopharyngolaryngoscopy was attempted, but the scope could not be negotiated toward the choanae. A No.6 nasogastric tube also could not be passed through the nostrils.

**Figure 1:**
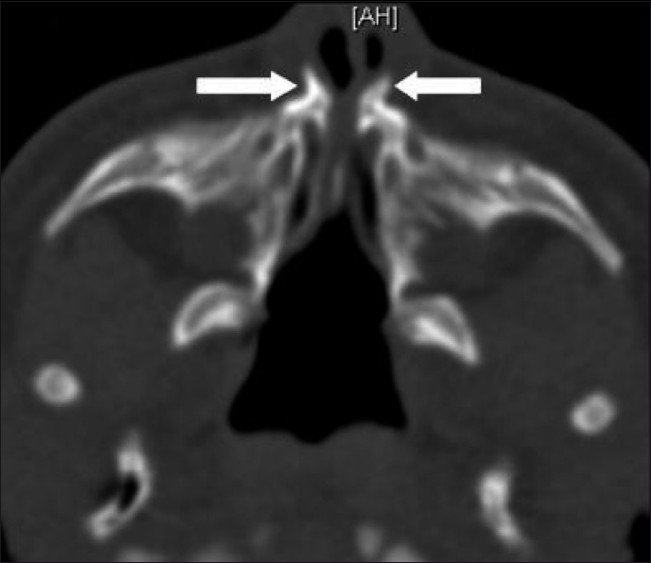
Axial CT scan shows medial approximation of the nasal processes of the maxilla (arrows) causing marked pyriform aperture narrowing

**Figure 2:**
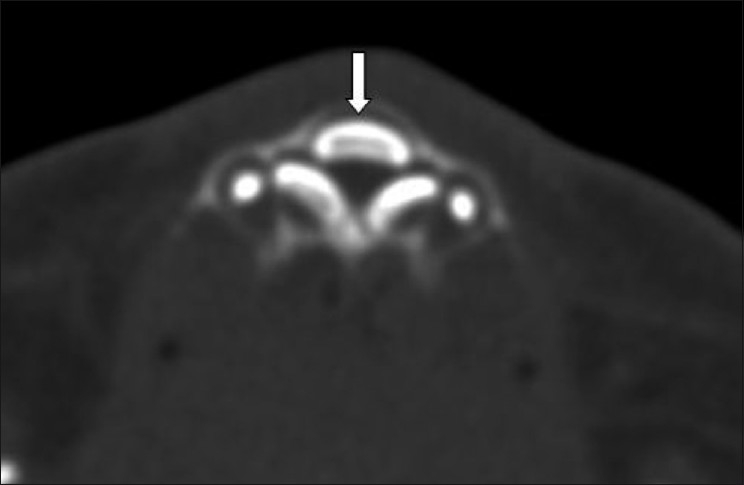
Axial CT scan shows an unerupted single central maxillary megaincisor (arrow)

**Figure 3:**
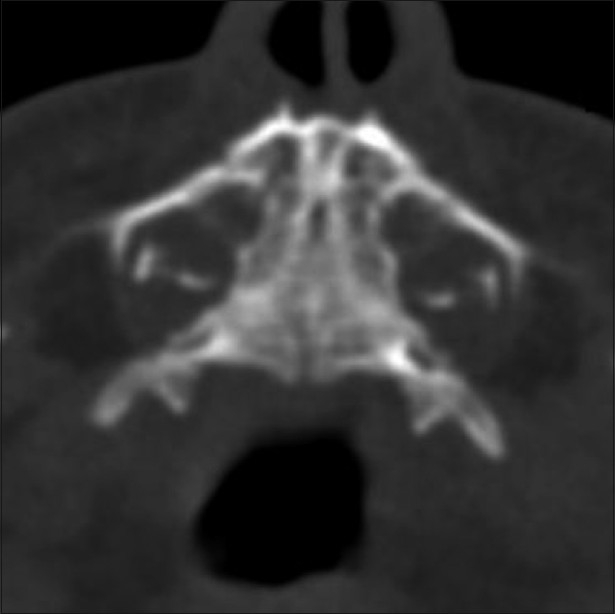
Axial CT scan shows a triangular hard palate

**Figure 4:**
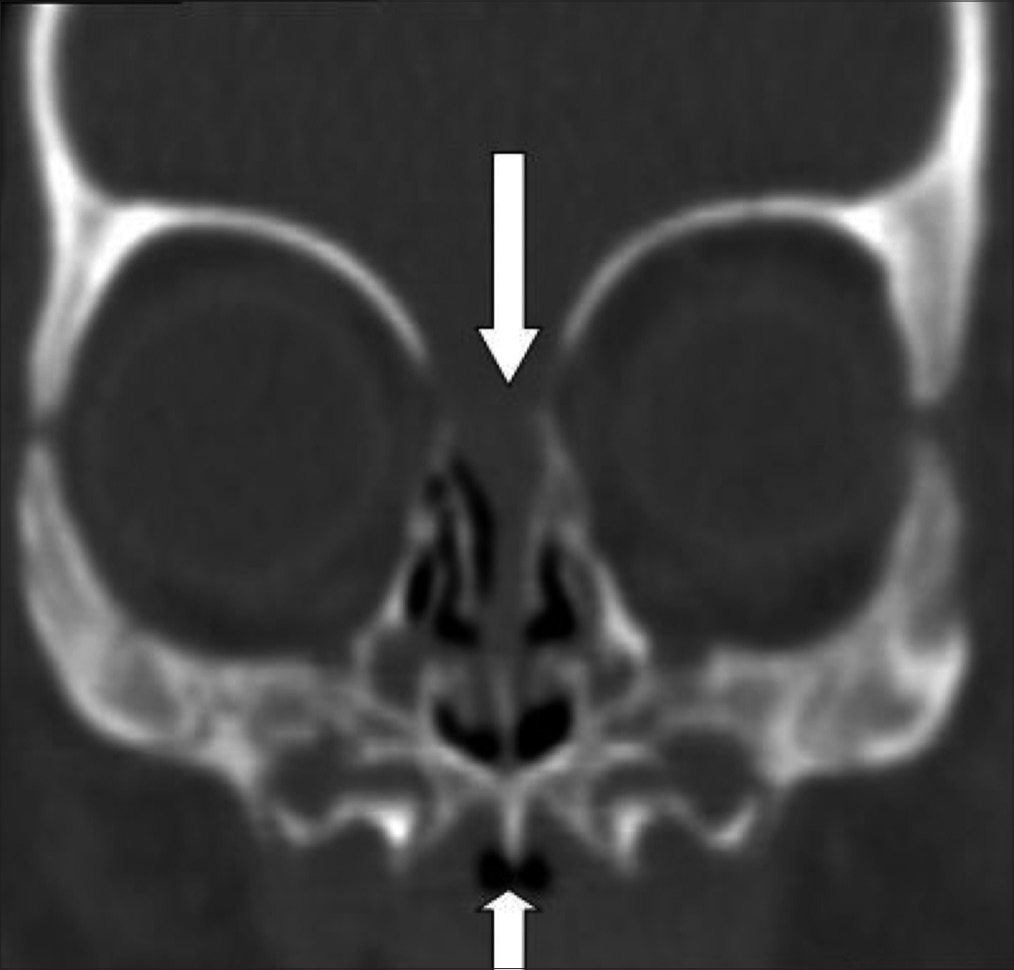
Coronal CT reconstruction shows a prominent inferior palatal ridge (short arrow) and a left nasoethmoid encephalocele (long arrow)

A CT scan was performed as the next step to evaluate the upper airways. This was negative for choanal atresia, but revealed multiple typical findings, which led to the diagnosis of CNPAS. The nasal cavity showed medial approximation of the nasal processes of the maxilla, causing marked narrowing of the pyriform apertures, which measured 3 mm in width on an axial image, at the level of the inferior meatus [Figure 1]. There was associated thinning of the anterior nasal septum. Additional findings included hypotelorism, a single maxillary central incisor tooth (megaincisor) [Figure 2], and a triangular hard palate [Figure 3] with a prominent median inferior palatal bony ridge [Figure 4]. The maxillary sinuses were hypoplastic. The perpendicular plate of the ethmoid and nasal bone was absent, as were the cribriform plate, the crista galli, and the fovea ethmoidalis on the left side, with a resultant nasoethmoid encephalocele in the superior left nasal cavity [Figure 4].

MRI was recommended to exclude known associated anomalies such as holoprosencephaly and anterior pituitary abnormalities, as well as for a detailed evaluation of the encephalocele. However, this was deferred to a follow-up visit. The child improved symptomatically with conservative measures such as insertion of an oral airway and feeding in the upright position. No active intervention was undertaken during this visit and the patient was asked to report for review earlier, in case there was any symptomatic worsening.

## Discussion

Nasal airway obstruction in newborns can lead to respiratory distress as they are obligatory nasal breathers. This is commonly caused by posterior choanal atresia. CNPAS is a rare cause of nasal airway obstruction in a neonate and occurs at a frequency of about one-fifth to one-third that of choanal atresia.[2]

The pyriform aperture (bony inlet) is the narrowest part of the normal nasal airway, and small changes in its cross-sectional area can result in a significant increase in nasal airway resistance.[4] CNPAS is characterized by the narrowing of the anterior bony nasal apertures. The clinical presentation can be variable, with respiratory distress at birth, cyclical cyanosis relieved by crying, or difficulty in breathing during feeding.[5]

**Figure 5:**
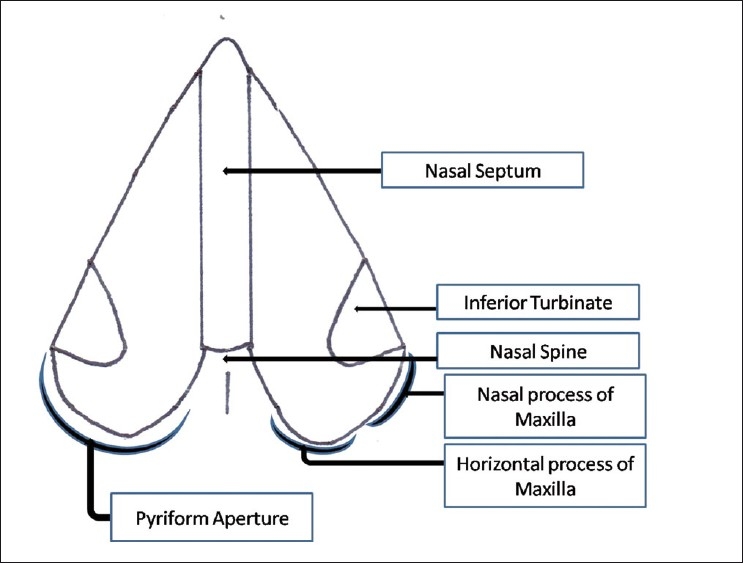
Schematic representation of the pyriform apertures in the coronal plane

Anatomically, the pyriform aperture is bounded laterally by the nasal processes of the maxilla and inferiorly by the junction of the horizontal processes of the maxilla [Figure 5]. The palate is formed from two primordia - the primary and secondary palates.[6] The primary palate is formed from the merging of the medial nasal prominences and becomes the premaxillary portion of the maxilla, which contains the incisor teeth. It represents only a small part of the adult hard palate, the os incisivum (anterior to the incisive foramen), and forms the floor of the pyriform apertures. The secondary palate develops from the lateral palatine processes of the maxilla, and gives rise to the hard and soft palates [located posterior to the incisive foramen].

Two theories about the pathogenesis of CNPAS exist: (a) deficiency of the primary palate, associated with a triangular hard palate, and (b) bony overgrowth in the nasal process of the maxilla, with a normal-shaped palate.[3] Our patient had a triangular hard palate, and in addition, exhibited a single central maxillary megaincisor [SCMI] and a prominent median inferior palatal bony ridge — these findings can be explained by the hypothesis of a primary palatal deficiency and strongly suggest the diagnosis of CNPAS.[2]

The diagnosis of pyriform aperture stenosis can be made accurately with a CT scan, by obtaining thin (1.5 to 3.0 mm), contiguous axial sections in a plane parallel to the anterior hard palate. It is important to demonstrate the narrowing on contiguous sections, as apparent narrowing may be caused by oblique imaging.[3] The normal range of width of the pyriform sinus in the age group of 0 – 6 months is 8.8 – 17.2 mm [median width = 13.5 mm].[7] Each pyriform aperture width less than 3 mm, or a whole pyriform aperture width less than 8 mm, in a term infant, confirms the diagnosis of CNPAS.[2]

There are two forms of CNPAS: an isolated form and a form that is associated with other anomalies including a midface dysostosis with associated central nervous system and endocrine abnormalities.[8] As SCMI has been recognized as a microform of holoprosencephaly, the presence of CNPAS along with a single megaincisor should prompt further evaluation with MRI, for possible holoprosencephaly and pituitary deficiency.[9–11] Once the diagnosis of CNPAS has been established, conservative treatment, which involves the use of topical nasal decongestants, humidification, insertion of oral airway, and lavage feeding, is the initial line of management. Surgical treatment aimed at widening the bony inlet via a sublabial approach, is performed only when this fails.[11] This is in contrast to the treatment of posterior choanal atresia, which is mainly surgical [dilatation and stenting, transpalatal repair, and transnasal resection using endoscopic sinus surgery techniques].[12]

This case has been presented to increase the awareness about this rare entity, to highlight the importance of recognizing the typical findings of CNPAS in cases undergoing evaluation of nasal airway obstruction, and the role of the CT scan in its diagnosis.
